# Tilden & Co.’s Alcoholic Extracts

**Published:** 1850-03

**Authors:** 


### TILDEN & CO.'S ALCOHOLIC EXTRACTS.

We have received, from the manufacturers of these extracts, several samples of the medicines, and from the trial we have made of them, we have no hesitation in pronouncing them superior to anything of the kind ever made in this country, Prof. Clark, one of the most reliable of men, in a letter to the editor of the New York Journal of Medicine, speaks in laudatory terms of the manufactory of Tilden & Co., after having visited the establishment. He says:

"I have lately visited the manufactory of these extracts. After inspecting the whole process, and examining a large number of preparations, I became so fully persuaded that these gentlemen have fallen upon the best plan of concentrating and preserving the active principles, especially of the narcotic vegetables, that I have voluntarily offered to them any assistance that I can render in introducing their medicines to the notice of the profession; being persuaded that these extracts must possess the efficiency and the uniformity of strength so necessary to the successful use of this class of remedies, and, I may add, so long sought for in vain.

"Should your conviction of the value of these preparations correspond with my own, after you have examined them and tried them in practice, perhaps you may think it due alike to the profession and to the gentlemen who are improving the instruments by which we work, to call the attention of your readers to the improvements which I cannot doubt this process secures."

We take great pleasure in commending Tilden & Co.'s extracts to the favor of the profession.

